# Electrophysiological Assessment of Serotonin and GABA Neuron Function in the Dorsal Raphe during the Third Trimester Equivalent Developmental Period in Mice^1^,^2^,^3^

**DOI:** 10.1523/ENEURO.0079-15.2015

**Published:** 2016-01-04

**Authors:** Russell A. Morton, Yuchio Yanagawa, C. Fernando Valenzuela

**Affiliations:** 1 Department of Neurosciences, University of New Mexico Health Sciences Center, Albuquerque, New Mexico 87131; 2 Department of Genetic and Behavioral Neuroscience, Gunma University Graduate School of Medicine, Maebashi 371-8511, Japan

**Keywords:** Monoamine, electrophysiology, neonatal

## Abstract

Alterations in the development of the serotonin system can have prolonged effects, including depression and anxiety disorders later in life. Serotonin axonal projections from the dorsal raphe undergo extensive refinement during the first 2 weeks of postnatal life in rodents (equivalent to the third trimester of human pregnancy). However, little is known about the functional properties of serotonin and GABA neurons in the dorsal raphe during this critical developmental period. We assessed the functional properties and synaptic connectivity of putative serotoninergic neurons and GABAergic neurons in the dorsal raphe during early [postnatal day (P) P5–P7] and late (P15–P17) stages of the third trimester equivalent period using electrophysiology. Our studies demonstrate that GABAergic neurons are hyperexcitable at P5–P7 relative to P15–P17. Furthermore, putative serotonin neurons exhibit an increase in both excitatory and GABA_A_ receptor-mediated spontaneous postsynaptic currents during this developmental period. Our data suggest that GABAergic neurons and putative serotonin neurons undergo significant electrophysiological changes during neonatal development.

## Significance Statement

During development, serotonin plays a major role in neuronal proliferation and differentiation, axonal migration, and synaptogenesis. However, the understanding of the functional development of this neurotransmitter system is limited. We characterized the functional properties of developing GABAergic and serotoninergic neurons in the dorsal raphe that together regulate the release 5-HT into many forebrain regions. Our studies indicate that both GABAergic and serotoninergic neurons undergo significant functional maturation during the first 2 postnatal weeks in mice. Therefore, alterations of the serotonin system induced by stress, drugs, or antidepressant agents during this critical period could have deleterious effects within the dorsal raphe as well as other brain regions innervated by serotoninergic axons.

## Introduction

Serotonin [5-hydroxytryptamine (5-HT)] belongs to the monoamine family of neurotransmitters and is synthesized from tryptophan by tryptophan hydroxylase. The 5-HT system is involved in the control of mood, arousal, and a number of cognitive processes. Alterations in this neurotransmitter system have been linked to several neuropsychiatric conditions, including depression, and anxiety disorders (Nikolaus et al., 2010; Ravindran and Stein, 2010; Lin et al., 2014; Olivier, 2015). Deficits in the interplay between 5-HT and other neurotransmitters, such as dopamine, play a role in the pathophysiology of schizophrenia, attention deficit hyperactivity disorder, and addiction (Kapur and Remington, 1996; Oades et al., 2008; Kirby et al., 2011). The cell bodies of 5-HT neurons are located in the raphe nuclei of the midbrain, pons, and medulla. Within the midbrain, these neurons can be found in the median and dorsal raphe (DR) nuclei, and their axons project to many brain regions, including the cerebral cortex, thalamus, and hippocampus (Jacobs and Azmitia, 1992). Local inhibitory GABAergic neurons regulate the excitability of DR 5-HT neurons and mediate feedforward inhibition driven by the prefrontal cortex (Celada et al., 2001). Importantly, this GABAergic modulation has been shown to play a role in the sleep–wake cycle (Gervasoni et al., 2000) and the acquisition of avoidance after social defeat (Challis et al., 2013). Furthermore, alterations in GABAergic signaling in the dorsal raphe play a role in anxiety induced by withdrawal from either cocaine or alcohol (Craige et al., 2015; Lowery-Gionta et al., 2015)

During development, 5-HT is a key modulator of neuron proliferation and differentiation, axon migration, and synaptogenesis (Frederick and Stanwood, 2009). Disruption of 5-HT levels or 5-HT receptor function during development causes emotional and cognitive deficits that last into adulthood. These deficits could be a consequence of abnormalities in neuronal circuitry assembly due to altered brain-derived neurotrophic factor levels (Vitalis et al., 2007; Lo Iacono and Gross, 2008; Migliarini et al., 2013; Witteveen et al., 2013; Donaldson et al., 2014; Suri et al., 2015). Studies with rodents have demonstrated that 5-HT neurons are born approximately on embryonic day 10 (E10), and begin to produce 5-HT between E12 and E13. 5-HT axons elongate between E13 and E16, and begin to reach their targets in the developing forebrain by E18 (Bonnin and Levitt, 2011). In human and nonhuman primates, these processes have been shown to occur during the first and second trimesters of pregnancy (Takahashi et al., 1986; Whitaker-Azmitia, 2001). During the first 2 weeks of life in rodents (equivalent to the third trimester of human pregnancy; Workman et al., 2013), 5-HT production dramatically increases and the majority of innervation of the forebrain by 5-HT axons occurs. However, the axonal refinement continues until postnatal day 21 (P21)(Lidov and Molliver, 1982; Lambe et al., 2000). The electrophysiological properties of dorsal raphe 5-HT neurons also change significantly during this period. Between P4 and P12, the membrane potential becomes more negative, the membrane resistance increases, action potential (AP) firing in response to current injection decreases, and excitatory and inhibitory synaptic currents begin to be detectable (Rood et al., 2014). These findings indicate that the 5-HT neurotransmitter system is significantly refined during the third trimester equivalent developmental period, making it particularly vulnerable to a variety of insults, such as exposure to substances of abuse (e.g., ethanol, cocaine), medications, and environmental toxins.

In this study, we investigated the electrophysiological properties of GABAergic neurons in comparison to putative 5-HT neurons in the DR at P5–P7 and P15–P17. Our data show that putative 5-HT neurons (in agreement with previously published data; Rood et al., 2014) and GABA neurons in the DR undergo significant electrophysiological changes during the first 2 weeks of postnatal life.

## Materials and Methods

### Animals and slice preparation

All animal procedures were performed in accordance with the authors’ University Institutional Care and Use Committees. Using the Venus fluorescent protein developed by Dr. Atsushi Miyawaki at RIKEN (Wako), Wang et al. (2009) generated the vesicular GABA transporter (VGAT)-Venus mouse line. We bred VGAT-Venus heterozygous females with wild-type C57BL6 males. Male mice were removed once pups were born. The VGAT-Venus pups were visually screened within the first 2 postnatal days using fluorescence-equipped goggles with an 480/40 nm excitation filter, and a long-pass 520 nm emission filter (BLS Ltd.). Both male and female pups were used for all experiments. For brain slice preparation, animals were heavily anesthetized with 0.75 g/kg ketamine followed by decapitation. Brain tissue was removed and incubated for 2–4 min in oxygenated ice-cold cutting solution containing the following (in mm): KCl, 2; NaH_2_PO_4_, 1.3; NaHCO_3_, 26; MgSO_4_, 12; CaCl_2_, 0.2; sucrose, 220; glucose, 10; and ketamine hydrochloride, 1 μg/ml. Coronal brainstem slices were generated using a vibrating slicer (1000 Plus Vibratome, Leica) at a thickness of 250 μm. Slices were incubated in oxygenated artificial cerebral spinal fluid (ACSF) containing the following (in mm): NaCl, 125; KCl, 2; NaH_2_PO_4_, 1.3; NaCO_3_, 26; glucose, 10, CaCl_2_, 2; MgSO_4_, 1 at 35°C for 40 min; and were allowed to recover at room temperature (21–22°C) for at least 30 min prior to recording. All chemicals were purchased from Sigma-Aldrich, unless otherwise specified.

### Electrophysiological recordings

Slices were maintained in ACSF during recording at ∼32°C. Neurons were morphologically identified by video monitoring of infrared differential interference contrast microscopy using an Olympus BX51WI upright microscope equipped with a metal oxide semiconductor digital camera (Model 01-ROL-BOLT-M-12, Q-Imaging) with a LUMPlan Fl/IR 40× water immersion lens 0.8 numerical aperture (NA; Olympus). VGAT-positive neurons were identified by Venus fluorescence using a mercury bulb, excitation filter ET470/40×, beam splitter T495lpxr, and emission filter ET525/50m (Chroma Technology Corp.). Patch pipettes were pulled from thin wall filament-containing borosilicate capillary glass with a P-97 Flaming/Brown Micropipette Puller (Sutter Instruments), with a resistance between 2 and 5 MΩ. Electrodes were filled with either K-gluconate (in mm: K-gluconate, 130; NaCl, 5; Na-phosphocreatine, 10; MgCl_2_, 1; HEPES, 10; EGTA, 0.02; MgATP, 2; NaGTP, 0.5; pH 7.3 with KOH) or KCl (in mm: KCl, 135; MgCl_2_, 2; EGTA, 0.5; HEPES, 10; Mg-ATP, 5; Na-GTP, 1; QX-314-Cl, 1; pH 7.25 with KOH) internal solutions. Recordings were obtained with a Multiclamp 700B amplifier and a Digidata 1440A. Data were acquired at 10 kHz with pClamp version 9 software and filtered at 1 kHz (Molecular Devices).

To measure current injection-induced action potential firing, the whole-cell patch-clamp configuration was used in the current-clamp mode. No current was injected to the cells to maintain a certain membrane potential. Current injections ranging from −40 to 100 pA were injected in 10 pA increments to induce hyperpolarization and depolarization. Action potential properties were measured from the first action potential triggered by the 70 pA current injection to avoid any confounding effects of adaptation. The change in voltage (ΔV) was calculated for each action potential, and we generated the phase plots by plotting ΔV/Δt versus membrane potential (in millivolts).

Spontaneous excitatory post-synaptic currents (sEPSCs) and GABA_A_ receptor-mediated postsynaptic currents (GABA_A_-PSCs) were recorded using the whole-cell patch-clamp configuration in the voltage-clamp mode. Excitatory currents were pharmacologically isolated using 10 µm gabazine [SR 955 31; 6-imino-3-(4-methoxyphenyl)-1(6H)-pyridazinebutanoic acid hydrobromide; Tocris Bioscience], and inhibitory currents were isolated using 50 µm dl-APV (dl-2-amino-7-phosphonovalerate, Tocris Bioscience; 1 mm kynurenic acid, Sigma-Aldrich), and 1 μm CPG 54626 [S-(R*,R*)]-[3-[[1-(3,4-dichlorophenyl)ethyl]amino]-2-hydroxypropyl](cyclohexylmethyl) phosphinic acid]. For each cell, the first 50 events were analyzed for each cell type at both ages using Mini-Analysis. Cumulative probability plots and averaged data was generated from each. The cumulative probability plots from all the cells of the same cell type and age were averaged together to generate the cumulative probability plots shown in the figures. The cumulative plots were analyzed using the Kolmogorov–Smirnov (K–S) test. The averaged data from each cell were combined to generate the bar graphs.

### Immunohistochemistry

Animals were anesthetized with isoflurane (Clipper Distribution Company) and perfused with ice-cold PBS containing heparin sodium 1 unit/L followed by ice-cold 4% paraformaldehyde in PBS. Brains were removed and immediately submerged in 4% paraformaldehyde, and incubated for 48 h at 4°C. After fixation, tissue was submerged in 30% sucrose in PBS until the tissue sank. Tissue was embedded in Optimal Cutting Temperature compound (Ted Pella) and flash frozen in 2-methyl butane cooled in a dry ice ethanol bath. Tissue was stored at −80°C, and 16-μm-thick coronal sections were cut using an HM505E cryostat (Microm International). Sections were stained with monoclonal anti-tryptophan hydroxylase (1:500; catalog #T0678, Sigma-Aldrich; Calizo et al., 2011; Zhao et al., 2011) primary antibody and goat anti-mouse IgG-conjugated to Alexa Fluor 555 (1:1000; catalog #A21434, Invitrogen) secondary antibody. DNA was labeled with NucRed live 647 (Molecular Probes) and were mounted using Fluoromount-G (Electron Microscopy Sciences). Low-magnification images were acquired using a BX51 upright microscope (Olympus) equipped with a 10× UPlanSApo/0.4 NA objective, mercury lamp, fluorescent filter sets [FITC/Chroma (catalog #39002), TRITC/Chroma (catalog #19004), Chroma Technologies] and an Olympus DP72 CCD camera. Confocal images were acquired using a 510 LSM inverted microscope (Carl Zeiss) equipped with an argon laser and an emission filter of 535/30 nm for green fluorescence detection. For red fluorescence images, a 543 nm HeNe laser was used with an emission filter of 605/55 nm. Far-red fluorescence was visualized with a 633 nm HeNe laser with a long-pass 560 nm emission filter. A 40× PlanApo/1.0 NA objective was used to collect images.

### Statistical analysis

All statistical analyses were performed with GraphPad Prism version 5.0. All datasets were tested for a normal distribution using the D’Agostino–Pearson test, and for outliers using the Rout test with *Q* = 1%. All statistical analyses of pooled data were performed using a two-tailed Student’s *t* test or Mann–Whitney tests, and the level of significance was considered to be *p* < 0.05. All statistics are given and data are presented as group means and SEMs. Action potential properties were measured using the action potential waveform 2 analysis utility in Mini-Analysis program. sEPSCs and GABA_A_-sPSCs were also analyzed with the Mini-Analysis program [including analysis by K–S test]. For details of results of statistical analyses, please see Table 1.

**Table 1:** Statistics

	Normal distribution?	Type of test	95% CI	Figure
a	No	Mann-Whitney test	−13.00 to 25.00	Figure 1G
b	Yes	Unpaired *t* test	−14.21 to 20.48	Figure 1I
c	Yes	Unpaired *t* test	−279.7 to 174.3	Figure 1H
d	Yes	Unpaired *t* test	−224.5 to 174.9	Figure 1J
e	Yes	Two-way ANOVA/Sidak test		Figure 2C
		0	−4.748 to 4.748	
		10	−4.748 to 4.748	
		20	−4.281 to 5.215	
		30	−3.008 to 6.488	
		40	−1.593 to 7.903	
		50	0.09514–9.591	
		60	1.460–10.96	
		70	1.515–11.01	
		80	1.362–10.86	
		90	2.179–11.67	
		100	3.075–12.57	
f	Yes	Two-way ANOVA/Sidak test		Figure 2D
		0	−7.016 to 7.016	
		10	−6.714 to 7.319	
		20	−6.151 to 7.881	
		30	−7.393 to 6.640	
		40	−7.934 to 6.099	
		50	−8.022 to 6.011	
		60	−7.914 to 6.119	
		70	−8.757 to 5.276	
		80	−8.829 to 5.204	
		90	−8.425 to 5.608	
		100	−8.268 to 5.765	
g	Yes	Unpaired *t* test	−13.78 to −1.867	Figure 2E
h	Yes	Unpaired *t* test	20.16–234.1	Figure 2F
i	Yes	Unpaired *t* test	−6.829 to 9.331	Figure 2G
j	No	Mann-Whitney test	−30.40 to 285.4	Figure 2H
k	Yes	Unpaired *t* test	−2.389 to 10.40	Figure 2I
l	Yes	Unpaired *t* test	−0.3530 to 0.01819	Figure 2J
	Yes	Unpaired *t* test	−0.7454 to 0.1780	Figure 2J
m	Yes	Unpaired *t* test	−4.370 to 8.690	Figure 2K
n	Yes	Unpaired *t* test	−0.6123 to −0.4160	Figure 2L
	Yes	Unpaired *t* test	−0.7372 to −0.2279	Figure 2L
o	Yes	Unpaired *t* test	−12.07 to 4.312	Figure 3E
p	Yes	Unpaired *t* test	−4.155 to 7.648	Figure 3F
q	Yes	Unpaired *t* test	−16.31 to 4.093	Figure 3G
r	Yes	Unpaired *t* test	−11.55 to 7.828	Figure 3H
s	Yes	Unpaired *t* test	−0.4426 to 0.2965	Figure 3I
t	No	Mann-Whitney test	−1.600 to 0.6000	Figure 3J
u	Yes	Unpaired *t* test	−7.116 to 8.198	Figure 3K
v	Yes	Unpaired *t* test	−8.008 to 5.609	Figure 3L
w	Yes	Unpaired *t* test	−443.2 to 415.8	Figure 4C
x	Yes	Unpaired *t* test	−335.8 to 754.5	Figure 4D
y	No	Mann-Whitney test	−15.70 to 3.638	Figure 4E
z	Yes	Unpaired *t* test	-33.27 to -5.591	Figure 4F
aa	Yes	Unpaired *t* test	−0.2000 to 0.3147	Figure 4G
bb	Yes	Unpaired *t* test	−0.07351 to 0.4604	Figure 4H
cc	Yes	Two-way ANOVA/Sidak test	tau1 2.265 to −2836	Figure 4I
			tau2 4.608 to −0.902	
dd	Yes	Two-way ANOVA/Sidak test	tau1 −0.043 to −1.006	Figure 4J
			tau2 −1.069 to −2.061	
ee	Yes	Unpaired *t* test	−999.6 to 170.1	Figure 5C
ff	Yes	Unpaired *t* test	−1916 to 205.6	Figure 5D
gg	Yes	Unpaired *t* test	−16.17 to 33.75	Figure 5E
hh	Yes	Unpaired *t* test	−70.48 to −1.596	Figure 5F
ii	Yes	Unpaired *t* test	−1.353 to −0.1316	Figure 5G
jj	No	Mann-Whitney test	−1.062 to 0.2104	Figure 5H
kk	Yes	Two-way ANOVA/Sidak test	tau1 −4.499 to 14.47	Figure 5I
ll			tau2 6.488–27.19	
ff2	Yes	Two-way ANOVA/Sidak test	tau1 −1.403 to 5.741	Figure 5J
gg1			tau2 0.3881–8.439	

## Results

### Distribution of GABAergic and serotonergic neurons in the dorsal raphe

**Figure 1.**
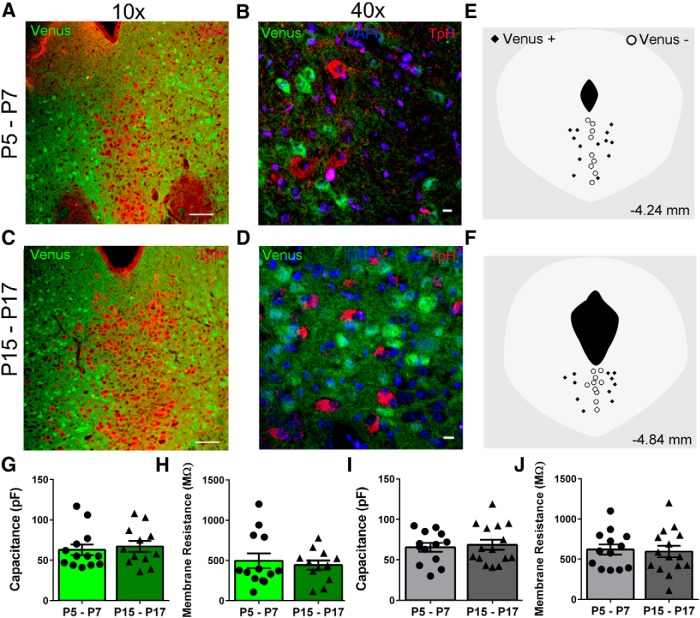
Distribution and passive membrane characteristics of Venus^+^ and Venus^−^ neurons. Immunohistochemistry of GABAergic neurons expressing Venus (green) and 5-HT neurons labeled with mouse anti-tryptophan hydroxylase (TpH; 1:500) and goat anti-mouse IgG-Alexa Fluor 555 antibodies (1:1000; red). ***A***, ***C***, Representative 10× images of the entire DR are shown for P5–P7 and P15–P17. Scale bar, 100 μm. ***B***, ***D***, Higher-magnification confocal images of boundary areas between midline and lateral areas taken from separate immunolabeled sections show that majority of neurons either express GABA (Venus, green) or serotonin (TpH, red; nuclei, blue). Scale bar, 10 μm. ***E***, ***F***, Representative locations of recordings from Venus^+^ and Venus^−^ neurons in sections approximately −4.24 mm (***E***) and −4.84 mm (***F***) away from bregma. ***G–J***, Membrane capacitance and resistance are shown for Venus^+^ (***G***, ***H***) and Venus^−^ (***I***, ***J***) neurons.

Serotonergic neurons receive inhibitory inputs from local GABAergic neurons (Liu et al., 1997) that are localized to the lateral portions of the DR (Gocho et al., 2013). Immunohistochemistry and fluorescence microscopy were used to assess the local distribution of serotonergic and GABAergic neurons in the DR during the third trimester equivalent developmental period. DR sections between −4.24 and −4.84 mm from bregma were obtained from P5–P7 and P15–P17 mice (Fig. 1*A–D*). GABAergic neurons were identified by Venus fluorescence and serotonergic neurons were immunolabeled with anti-tryptophan hydroxylase. The anatomical distribution of both serotonergic and GABAergic neurons was similar to that previously reported (Calizo et al., 2011; Gocho et al., 2013; Rood et al., 2014; Corteen et al., 2015). Serotonergic neurons were localized to the medial and lateral wings of the DR, whereas the GABAergic neurons were localized to the lateral portions of the DR (Fig. 1*A*,*C*). Higher-magnification confocal images show that there was no overlap between the Venus and tryptophan hydroxylase fluorescence at either age (Fig. 1*B*,*D*).

Serotonergic neurons located within different subfields of the DR have been shown to be electrophysiologically and morphologically distinct (Calizo et al., 2011), especially during this development period (Rood et al., 2014). Our studies focused on GABAergic neurons (i.e., Venus^+^) and putative 5-HT neurons (i.e., Venus^−^) located in and around the ventral medial subfield of the DR. Representative recording positions for both Venus^+^ and Venus^−^ neurons in the DR are shown in Figure 1, *E* and *F*. Passive membrane properties (membrane capacitance and resistance) were recorded with whole-cell patch-clamp electrophysiology from both Venus^+^ and Venus^−^ neurons. The membrane capacitance was similar in Venus^+^ and Venus^−^ neurons (∼65 pF), and did not significantly change with age (Fig. 1*G* ^a^: Venus^+^, Mann-Whitney *U* test = 67.50, *n* = 12 neurons from 7 animals, *p* = 0.5828; and Fig. 1*I* ^b^: Venus^−^, *t*_(26)_ = 0.3719, *p* = 0.7130, *n* = 15 neurons from 7 animals). Membrane resistance was also similar at the two ages in both Venus^+^ and Venus^−^ neurons (Fig. 1*H* ^c^: Venus^+^, *t*_(23)_ = 0.4804, *p* = 0.6355; *n* = 12 neurons from 7 animals; and Fig. 1*J* ^d^: Venus^−^, *t*_(26)_ = 0.2550, *p* = 0.8007, *n* = 15 neurons from 7 animals).

### Excitability of Venus^+^ and Venus^−^ neurons

**Figure 2.**
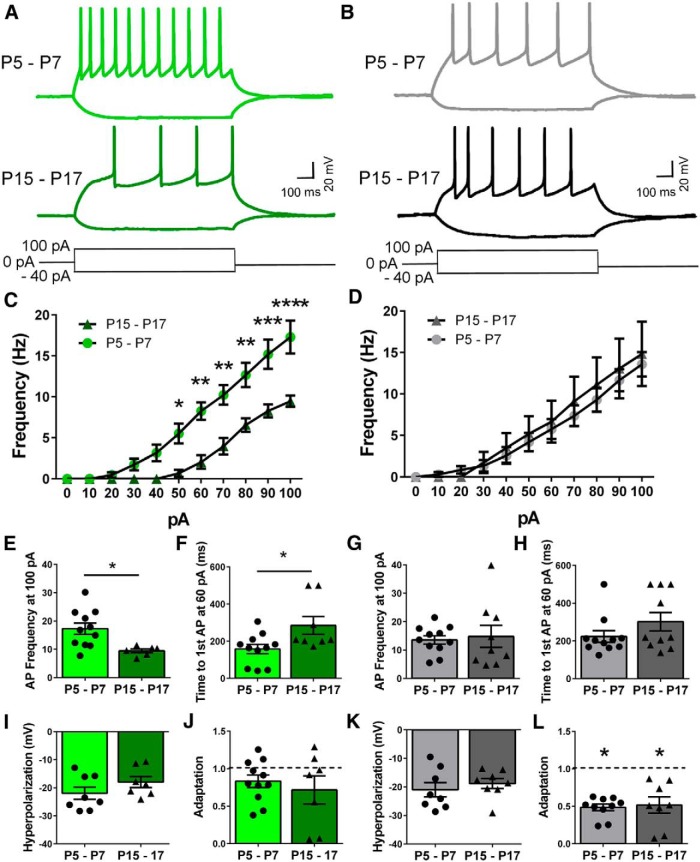
Excitability of Venus^+^ and Venus^−^ neurons. ***A***, ***B***, Representative traces of hyperpolarizing and depolarizing potentials are shown for Venus^+^ (***A***) and Venus^−^ (***B***) neurons at both ages elicited by either a −40 or 100 pA current injection. ***C***, ***D***, Giving sequential current injections and recording the action potential frequency, we generated input–output curves for both Venus^+^ (***C***) and Venus^−^ (***D***) neurons at the indicated age ranges (two-way ANOVA/Sidak test, *p < 0.05, **p < 0.01, ***p < 0.001, ****p < 0.0001). ***E–H***, Excitability was assessed by measuring the action potential firing frequency with 100 pA injected and the time to the first action potential when 60 pA was injected in both Venus^+^ (***E***, ***F***) and Venus^−^ (***G***, ***H***) neurons. ***I***, ***K***, Hyperpolarization was measured as the negative peak membrane potential evoked by −40 pA injection in Venus^+^ (***I***) and Venus^−^ (***K***) neurons at both ages. ***J***, ***L***, Adaptation was measured by dividing the instantaneous frequency of the last two action potentials by the instantaneous frequency of the first two action potentials when 100 pA was injected into Venus^+^ (***J***) and Venus^−^ (***L***) neurons at both ages.

Active membrane properties of both Venus^+^ and Venus^−^ neurons were examined using whole-cell current-clamp electrophysiology with no current injected at baseline. The resting membrane potential did not significantly change during this developmental period in Venus^−^ (P5–P7 = −57.02 ± 5.5 mV vs P15–P17 = −57.01 ± 5.8 mV, *t*_(19)_ = 0.001) or Venus^+^ (P5–P7 = −55.87 ± 4.8 mV vs P15–P17 = −60.71 ± 2.7 mV, *t*_(17)_ = 0.780) neurons. Representative traces of events triggered by hyperpolarizing and depolarizing current injections are shown in Figure 2, *A* and *B*. Input–output plots were generated by graphing the current injections from 0 to 100 pA in 10 pA steps versus the action potential frequency for each cell type at P5–P7 and P15–P17 (Fig. 2*C* ^e^,*D* ^f^). Venus^+^ neurons from the P5–P7 group fired at a higher frequency than those from the P15–P17 group (Fig. 2*C*, two-way ANOVA: interaction *F*_(10,150)_ = 6.685, *p* < 0.0001; current injected *F*_(10,150)_ = 74.14, *p* < 0.0001; age (*F*_(1,150)_ = 10.22, *p* = 0.006; Sidak *post hoc* test, *p* <0.05 at 50–100 pA). However, this effect was not observed in Venus^−^ neurons (Fig. 2*D*; two-way ANOVA: interaction, *F*_(10,180)_ = 0.2960, *p* = 0.9814; current injected, *F*_(910,180)_ = 41.95, *p* < 0.0001; age *F*_(1,18)_ = 0.1495, *p* = 0.7036; Sidak *post hoc* test, *p* > 0.05). Venus^+^ neurons from P5–P7 animals fired at a higher frequency than did those from P15–P17 animals when a current injection of 100 pA was applied (Fig. 2*E* ^g^; *t*_(15)_ = 2.800, *p* = 0.0135, *n* = 11 neurons from 7 animals), and time to the first action potential was shorter in P5–P7 animals (Fig. 2*F* ^h^; *t*_(17)_ = 2.508, *p* = 0.0226, *n* = 11 neurons from 7 animals). Neither the firing frequency at 100 pA nor the time to the first action potential at 60 pA were significantly different in Venus^−^ neurons from P5–P7 versus P15–P17 mice (Fig. 2*G* ^i^: *t*_(18)_ = 0.3254, *p* = 0.7486, *n* = 8 neurons from 5 animals; and Fig. 2*H* ^j^: Mann-Whitney *U* test = 38.50, *p* = 0.2578, *n* = 8 neurons from 5 animals). A current injection of −40 pA caused similar membrane potential hyperpolarization regardless of cell type or animal age (Fig. 2*I* ^k^: Venus^+^, *t*_(13)_ = 1.353, *p* = 0.1991, *n* = 8 neurons from 5 animals; and Fig. 2*K* ^m^: Venus^−^, *t*_(14)_ = 0.7094, *p* = 0.4897, *n* = 8 neurons from 5 animals). Spike-firing adaptation is an intrinsic property of some neurons that reduces the frequency of action potentials upon sustained depolarization. To investigate adaptation in our experiments, we divided the instantaneous frequency between the last two action potentials by the instantaneous frequency between the first two action potentials when a current injection of 100 pA was applied. Adaptation was determined to be present if the instantaneous frequency ratio was significantly different from 1. Venus^+^ neurons overall did not display significant firing rate adaptation (Fig. 2*J* ^l^: P5–P7, *t*_(10)_ = 2.010, *p* = 0.0722; P15–P17, *t*_(6)_ = 1.504, *p* = 0.1834, *n* = 6 neurons from 4 animals). Serotonergic neurons are known to display adaptation that is dependent upon the inactivation rate of voltage-gated sodium channels (Milescu et al., 2010). Venus^−^ neurons at both age groups displayed significant adaptation (Fig. 2*L* ^n^: P5–P7, *t*_(9)_ = 11.85, *p* < 0.0001, *n* = 9 neurons from 7 animals; P15–P17, *t*_(7)_ = 4.481, *p* = 0.003, *n* = 7 neurons from 4 animals).

### Action potential characterization

**Figure 3.**
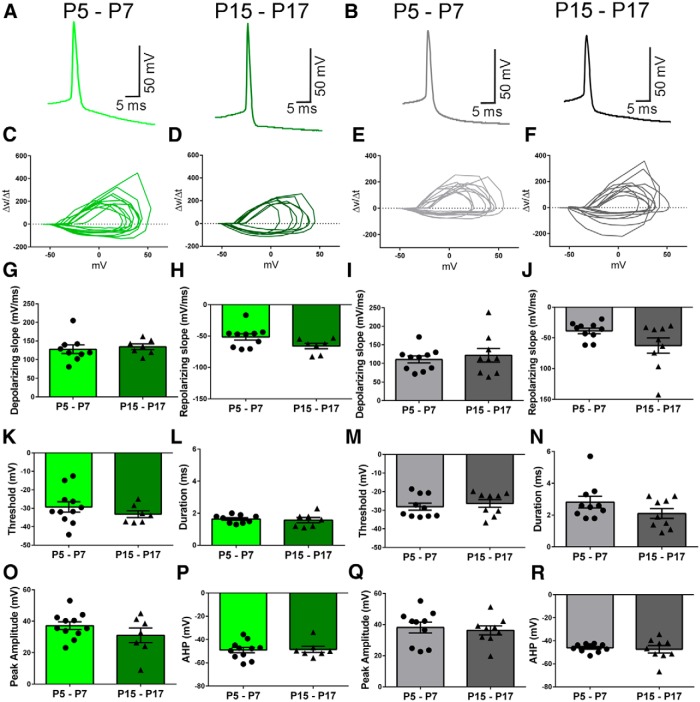
Action potential properties of Venus^+^ and Venus^−^ neurons. The first action potential that was generated from the 70 pA current injection from each cell was then averaged together to generate the action potential traces. ***A***, ***B***, The averaged action potentials traces are shown for Venus^+^ (***A***) and Venus^−^ (***B***) neurons at P5–P7 and P15–P17. ***C–F***, We generated the phase plots by comparing the ΔV/Δt versus the membrane potential (mV) for each Venus^+^ (***C***, ***D***) or Venus^−^ (***E***, ***F***) neuron at both ages. ***G–J***, We also measured the depolarizing and repolarizing slopes of the action potentials from Venus + (***G***, ***H***) and Venus^−^ (***I***, ***J***) neurons. The individual action potentials were used to measure the action potential properties. ***K–R***, We used the Mini-Analysis software AP waveform analysis 2 to measure the action potential threshold (***K***, ***M***), duration (***L***, ***N***), peak amplitude (***O***, ***Q***), and AHP (***P***, ***R***).

We analyzed the properties of individual action potentials by examining the first action potential generated by a 70 pA current injection to avoid any confounding effects of adaptation. The first action potential triggered by the 70 pA current injection for each cell was averaged together to generate the traces shown in Figure 3, *A* and *B*, for both Venus^+^ and Venus^−^ neurons at each age. We generated phase plots by plotting the ΔV/Δt versus the membrane potential (mV). Each line represents individual action potentials from Venus^+^ or Venus^−^ neurons at either age (Fig. 3*C–F*). Both the depolarizing and repolarizing slopes were measured for each individual cell (Fig. 3*G–J*). Again, using the first action potential triggered by the 70 pA current injection from each cell, we measured the action potential threshold, peak amplitude, duration, and afterhyperpolarization (AHP). The action potential threshold (Fig. 3*K* ^o^: Venus^+^, *t*_(16)_ = 1.004, *p* = 0.3304, *n* = 11 neurons from 7 animals; and Fig. 3*M* ^p^: Venus^−^, *t*_(17)_ = 0.6243, *p* = 0.5407, *n* = 11 neurons from 7 animals) and action potential duration (Fig. 3*L* ^s^: Venus^+^, *t*_(14)_ = 0.4238, *p* = 0.6782, two outliers removed; Fig. 3*N* ^t^: Venus^−^, Mann-Whitney *U* test = 30, *p* = 0.2341) did not differ between cell types or age, and neither did the peak amplitude (Fig. 3*O* ^q^: Venus^+^, *t*_(16)_ = 1.270, *p* = 0.2224; Fig. 3*Q* ^r^: Venus^−^, *t*_(17)_ = 0.4051, *p* = 0.6904, *n* = 11 neurons from 7 animals) or AHP (Fig. 3*P* ^u^: Venus^+^, *t*_(16)_ = 0.1497, *p* = 0.8829; Fig. 3*R* ^v^: Venus^−^, *t*_(17)_ = 0.3717, *p* = 0.7147) between ages.

### Spontaneous EPSCs

**Figure 4.**
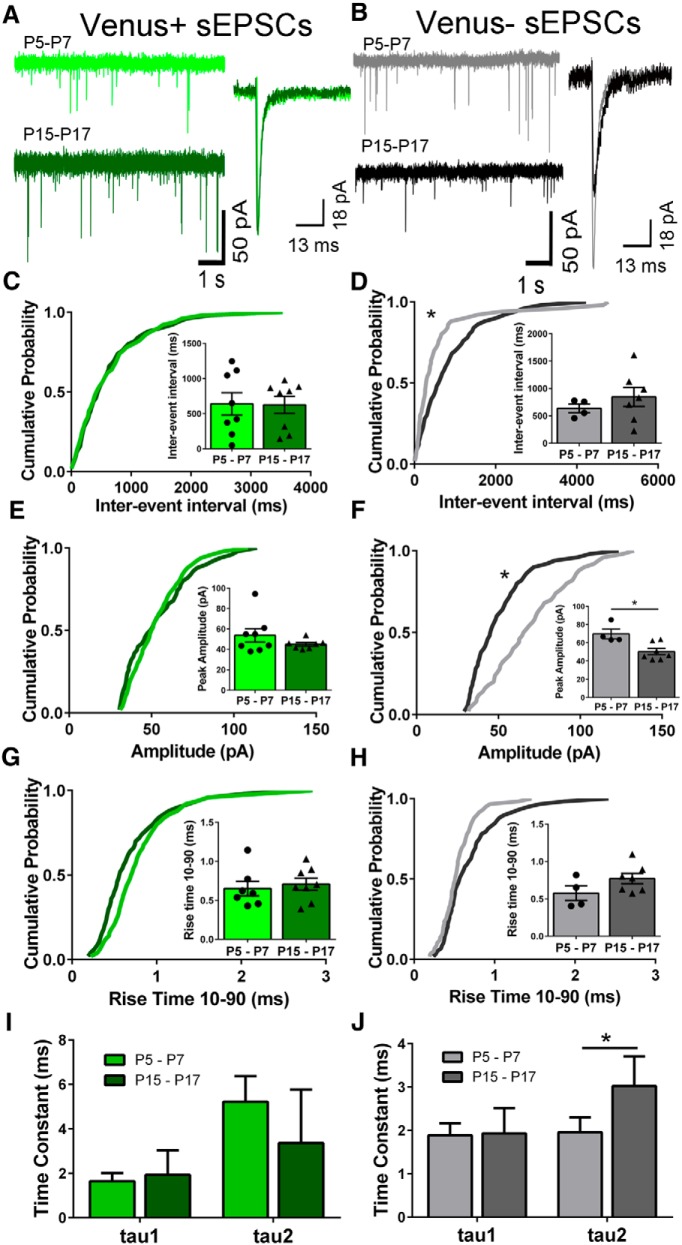
sEPSCs in Venus^+^ and Venus^−^ neurons. ***A***, ***B***, Representative currents and averaged currents from individual cells are shown for both Venus^+^ (***A***) and Venus^−^ (***B***) neurons. The cumulative probability plots from all the cells were averaged together to generate the cumulative probability plots shown for each parameter. Pooled data are shown in the inset bar graph. ***C–H***, The interevent interval (***C***, ***D***), peak amplitude (***E***, ***F***), and rise time (***G***, ***H***) are shown as a cumulative probability plot, and averaged data in the inset bar graphs for both Venus^+^ and Venus^−^ neurons. ***I***, ***J***, The averaged sEPSC for each cell was fitted with a double exponential function to calculate the decay rates for Venus^+^ (***I***) and Venus^−^ (***J***) neurons.

We examined sEPSCs in Venus^+^ and Venus^−^ neurons at P5–P7 and P15–P17. Representative traces of sEPSCs from individual cells are shown for Venus^+^ and Venus^−^ neurons at both ages, with the average sEPSCs for those same cells being shown in the right panel (Fig. 4*A*,*B*). To assess sEPSCs, we generated both cumulative plots from the average of all recordings and bar graphs of pooled data obtained from all the cells. In Venus^+^ neurons, the sEPSC interevent interval, (Fig. 4*C* ^w^; K–S test, *D* = 0.06, *p* > 0.9999; *t*_(14)_ = 0.06833, *p* = 0.9465, *n* = 16 neurons from 5 animals), amplitude (Fig. 4*E* ^y^; K–S test, *D* = 0.12, *p* = 0.8643; ^y^Mann-Whitney *U* test = 22, *p* = 0.5358, *n* = 16 neurons from 5 animals), or rise time (Fig. 4*G* ^aa^; K–S test, *D* = 0.22, *p* = 0.1777; *t*_(13)_ = 0.4816, *p* = 0.6381, one outlier removed, *n* = 16 neurons from 5 animals) did not differ between ages. Venus^−^ neurons displayed a significant left shift in the cumulative probability distribution of the interevent interval, but there was no significant difference in the averaged data (Fig. 4*D* ^x^; K–S test, *D* = 0.28, *p* = 0.0397; *t*_(9)_ = 0.8686, *p* = 0.4076, *n* = 11 neurons from 5 animals). In the Venus^−^ neurons, the cumulative probability distribution of the peak amplitude for the P15–P17 group was left shifted in comparison to that of the P5–P7 group. Analysis of the averaged date revealed a significant decrease in the sEPSC peak amplitude (Fig. 4*F* ^z^; K–S test, *D* = 0.38, *p* = 0.0015; *t*_(9)_ = 3.176, *p* = 0.0113, *n* = 11 neurons from 5 animals). The sEPSCs rise time was not significantly different between the two ages in the Venus^+^ or Venus^−^ neurons (Fig. 4*H* ^bb^; K–S test, *D* = 0.2203, *p* = 0.114; *t*_(9)_ = 1.639, *p* = 0.1356, *n* = 11 neurons from 5 animals). Average traces of sEPSCs recorded from an individual cell were used to calculate EPSC decay using a double exponential function. In Venus^+^ neurons, the decay constants did not significantly change between the two ages (Fig. 4*I* ^cc^; two-way ANOVA: interaction, *F*_(1,26)_ = 1827, *p* = 0.1881; tau, *F*_(1,26)_ = 10.02, *p* = 0.0039; age, *F*_(1,26)_ = 0.9819, *p* = 0.3309, 2 outliers removed; Sidak test, *p* > 0.05). In Venus^−^ neurons, there was no significant interaction but tau2 was significantly longer in the older animals (Fig. 4*J* ^dd^; interaction: *F*_(1,17)_ = 3.312, *p* = 0.0864; tau, *F*_(1,17)_ = 4.241, *p* = 0.0551; age: *F*_(1,17)_ = 3.886, *p* = 0.0652; 1 outlier removed, Sidak test; tau1: *t*_(17)_ = 0.1086, *p* = 0.9927; tau2: *t*_(17)_ = 2.642, *p* = 0.0339).

### Spontaneous GABA_A_ receptor-mediated postsynaptic currents

**Figure 5.**
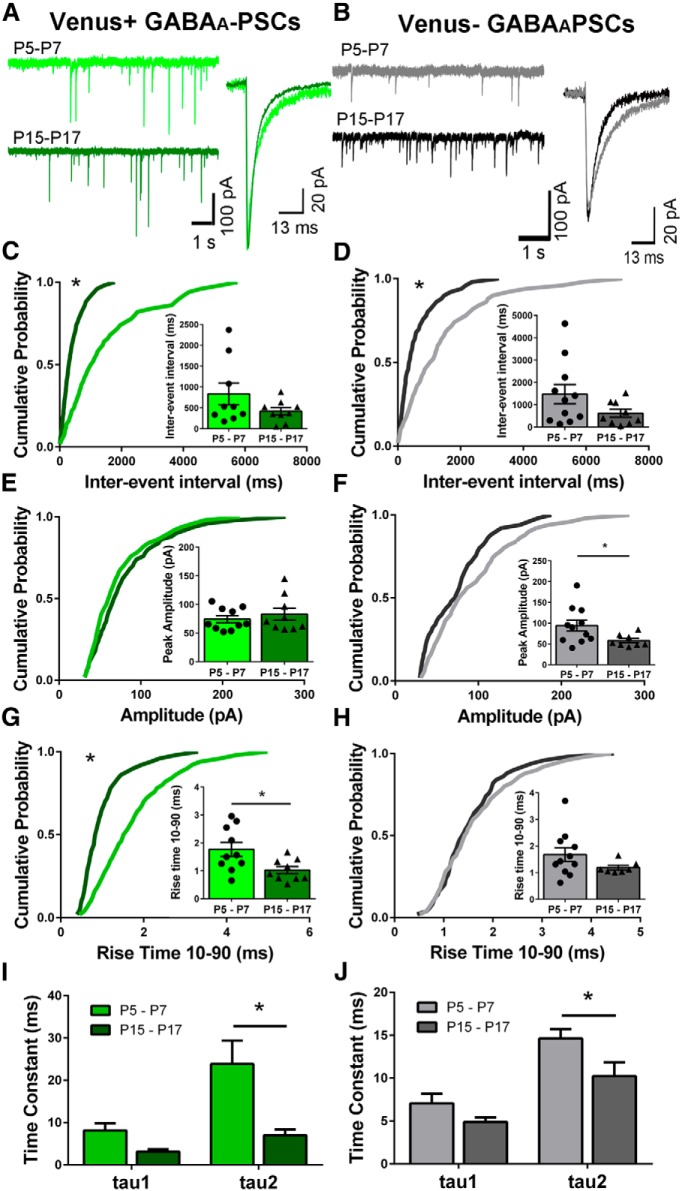
GABA_A_-sPSCs in Venus^+^ and Venus^−^ neurons. ***A***, ***B***, Representative traces and averaged currents from individual cells are shown for Venus^+^ (***A***) and Venus^−^ (***B***) neurons. The cumulative probability plots from all the cells were averaged together to generate the cumulative probability plots shown for each parameter. Pooled data are shown in the inset bar graph. ***C–H***, The interevent interval (***C***, ***D***), peak amplitude (***E***, ***F***), and rise time (***G***, ***H***) are shown as a cumulative probability plot, and averaged data are shown in the inset bar graphs for both Venus^+^ and Venus^−^ neurons. ***I***, ***J***, The averaged GABA_A_-PSC for each cell was fitted with a double exponential curve to calculate the decay rates for Venus^+^ (***I***) and Venus^−^ (***J***) neurons.

We next examined GABA_A_-PSCs in both cell types. Representative traces from individual cells are shown for both Venus^+^ (Fig. 5*A*) and Venus^−^ (Fig. 5*B*) neurons at P5–P7 and P15–P17 along with the averaged current trace of that same cell in the right-hand panel. Both Venus^+^ and Venus^−^ neurons show a significant leftward shift in the older animals. However, analysis of the bar graph did not reveal a significant difference between age groups [Venus^+^: (Fig. 5*C* ^ee^) K–S test, *D* = 0.48, *p* < 0.0001; *t*_(16)_ = 1.503, *p* = 0.1522, one outlier removed, *n* = 19 neurons from 10 animals; Venus^−^: (Fig. 5*D* ^ff^) K–S test, *D* = 0.34, *p* = 0.0062; *t*_(18)_ = 1.694, *p* = 0.1075, *n* = 20 neurons from 12 animals]. The cumulative probability distributions of GABA_A_-PSC peak amplitudes were similar in both age groups in Venus^+^ neurons (Fig. 5*E* ^gg^; K–S test, *D* = 0.12, *p* = 0.8643; *t*_(17)_ = 0.7429, *p* = 0.4677, *n* = 19 neurons from 10 animals). However, we did not find a significant difference in the cumulative probability plots, but analysis of the bar graph revealed a significant decrease in event amplitude in Venus^−^ neurons (Fig. 5*F* ^hh^; K–S test, *D* = 0.18, *p* = 0.3927; *t*_(17)_ = 2.208, *p* = 0.0413, one outlier removed, *n* = 19 neurons from 12 animals). The GABA_A_-PSCs in Venus^+^ neurons displayed a faster rise time in the older animals (Fig. 5*G* ^ii^; K–S test, *D* = 0.44, *p* = 0.0001; *t*_(17)_ = 2.564, *p* = 0.0201, *n* = 19 neurons from 10 animals). Conversely, the rise time was similar in Venus^−^ neurons from both age groups (Fig. 5*H* ^jj^; K–S test, *D* = 0.1, *p* = 0.9639; Mann-Whitney *U* test, *D* = 25, *p* = 0.2463, one outlier removed, *n* = 20 neurons from 12 animals). The GABA_A_-PSCs recorded from individual cells were averaged and fitted to a double exponential function to calculate the decay time constants for Venus^+^ and Venus^−^ neurons at both ages. For both Venus^+^ and Venus^−^ neurons, the tau2 time constant was reduced in older animals [Venus^+^: (Fig. 5*I* ^kk^), interaction, *F*_(1,29)_ = 3.963, *p* = 0.056; tau, *F*_(1,29)_ = 10.79, *p* = 0.0027; age, *F*_(1,29)_ = 13.44, *p* = 0.001; three outliers removed; tau1, *t*_(29)_ = 1.24, *p* > 0.05; tau2, *t*_(29)_ = 3.837, *p* < 0.05); Venus^−^: (Fig. 5*J* ^ll^), interaction, *F*_(1,28)_ = 0.9709, *p* = 0.3329; tau, *F*_(1,28)_ = 32.04, *p* < 0.0001; age, *F*_(1,28)_ = 8.352, *p* = 0.0074, five outliers removed; *t*_(18)_ = 2.591, *p* < 0.05; tau1, *t*_(28)_ = 1.435, *p* > 0.05; tau2, *t*_(28)_ = 2.591, *p* <0.05).

## Discussion

Our studies demonstrate that DR GABAergic neuron excitability decreases as the frequency of GABA_A_-PSCs increases during the third trimester equivalent. At P15–P17, putative 5-HT neurons exhibit an increased frequency of both sEPSCs and GABA_A_-sPSCs. Together, these data highlight the important developmental processes that take place in the DR during this period of development.

GABAergic neurons and putative 5-HT neurons coexist in the DR and undergo distinct changes in excitability during the third trimester equivalent.

We found that the vast majority of GABAergic neurons are located in the lateral regions of the DR, whereas 5-HT neurons are located predominantly in the medial region of the DR. However, we also observed scattered Venus^+^ GABAergic neurons and tryptophan hydroxylase^+^ 5-HT neurons near midline and lateral areas, respectively. Furthermore, the distribution of these neurons was not different in sections from P5–P7 and P15–P17 mice, and we did not detect any neurons that coexpressed Venus and tryptophan hydroxylase at these developmental periods. These findings are in agreement with those of Gocho et al. (2013), who found a similar distribution of 5-HT and GABAergic neurons in the DR of adolescent mice expressing green fluorescence protein driven by the glutamate decarboxylase 67 (GAD67) promoter. Our results are also generally consistent with those of Rood et al. (2014), who detected scattered clusters of neurons that stained positive for GAD67 in the lateral portion of the ventromedial DR of P4 mice, as well as in the vicinity of 5-HT neurons located in the midline. It should be noted that the density of GABAergic neurons in our DR sections from P5–P7 VGAT-Venus mice appears to be higher than the density of these neurons in the P4 Pet-1-YFP mice used by Rood et al. (2014). Potential explanations for this difference include the following: there are strain differences in the number of DR GABAergic neurons present during the first week of neonatal development; the number of DR GABAergic neurons significantly increases at P5–P7 with respect to P4; or more GABAergic neurons can be detected using mice expressing Venus protein than using antibodies against endogenous GAD67. Regarding the GAD67 immunoreactivity, it is possible that a significant population of GABAergic neurons may not have been detected at P4 because they express low levels of GAD67.

We compared the functional properties of Venus^+^ and Venus^−^ neurons in the DR in neonatal mice. Venus^+^ can be unambiguously defined as GABAergic neurons because the expression of this fluorescent protein is driven by VGAT. Venus^−^ neurons likely correspond to 5-HT neurons because their electrophysiological properties are consistent with those previously described using mice expressing YFP (yellow fluorescent protein) under the control of the 5-HT neuron-specific Pet-1 promoter (Pet-1::YFP; Rood et al., 2014). Specifically, Venus^−^ cells exhibit similar resting membrane potentials, membrane resistance, action potential threshold, action potential duration, AHP, and action potential adaptation (Milescu et al., 2010; Calizo et al., 2011). However, given that we cannot eliminate the possibility that some of the Venus^−^ cells that we recorded from are of other neuronal subtypes (e.g., glutamatergic), we cautiously refer to these as putative 5-HT neurons.

We found a striking difference in the excitability of GABAergic neurons between P5–P7 and P15–P17. Specifically, GABAergic neurons from P5–P7 mice fired action potentials at a lower threshold and at a higher frequency in response to current injections. The hyperexcitability was not due to changes in membrane resistance. Redistribution of voltage-gated sodium channels from the axon hillock to the soma has been shown to reduce the firing threshold in developing cortical and dentate granule neurons (Cummins et al., 1994; Kress et al., 2010). The redistribution of voltage-gated sodium channels could explain the firing of APs with smaller current injections and decreased time to the first AP without changing the AP threshold. It is also possible that changes in *I*_A_ (A type) currents may play a role in these developmental changes (Gorter et al., 1995).

In contrast to Venus^+^ GABAergic neurons, we found that Venus^−^ putative 5-HT neurons fired action potentials in response to current injection with a similar frequency at both ages. Consistent with this, we did not observe any age-dependent differences in membrane resistance or resting membrane potential. These findings are different from those of Rood et al. (2014), who showed with Pet-1-YFP mice that the excitability of 5-HT neurons in the ventromedial DR is reduced at P12 and P21 with respect to P4, which may be partially due to a shift to a more hyperpolarized membrane potential at the older ages. A potential explanation for the differences between these studies is that the reduction in putative 5-HT neuron excitability and the membrane potential hyperpolarization take place relatively rapidly, between P4 and P5, reaching a stable level between P5 and P7. Alternatively, it is possible that the functional maturation of 5-HT neurons follows a different developmental time course in VGAT-Venus and Pet-1-YFP mice. However, all other parameters we measured at the two ages, such as action potential threshold, action potential peak amplitude, AHP amplitude, and action potential duration are in general agreement with those reported by Rood et al. (2014).

Adaptation of action potential frequency is an intrinsic property of 5-HT neurons (Kirby et al., 2003) and is mediated by the inactivation of Na^+^ channels (Milescu et al., 2010) rather than activation of BK/SK channels, Ca^2+^-dependent K^+^ channels, or K_v_7/M channels, which participate in this process in neurons from several other brain regions (Madison and Nicoll, 1984; Storm, 1990; Gu et al., 2005; Nigro et al., 2014). Spike–frequency adaptation has been used for the electrophysiological identification of 5-HT neurons, and is thought to play a role in signal integration and self-inhibition (Vandermaelen and Aghajanian, 1983). Our data show that putative 5-HT neurons exhibit significant adaptation as early as P5–P7 and that the magnitude of this phenomenon does not change by P15–P17. Overall, Venus^+^ neurons did not display significant adaptation at the current injections we used. However, in our studies a few GABA neurons at P15–P17 did display significant adaptation. Furthermore, Gocho et al. (2013) showed that larger current injections in older animals resulted in significant adaptation. Adaptation is one of many firing characteristics of GABA neurons from other brain regions, including the cortex and hippocampus (Ali et al., 1998; Stiefel et al., 2013).

### Changes in synaptic currents in GABAergic neurons and putative 5-HT neurons

Rood et al. (2014) found that sEPSC frequency gradually increased with age in 5-HT neurons of the ventromedial and lateral wing regions of the DR of Pet-1-YFP mice. Specifically, sEPSC frequency increased by approximately five fold between P4 and P21 in 5-HT neurons of the ventromedial DR without a significant change in amplitude or decay. This finding could be interpreted to reflect the integration of 5-HT DR neurons into the glutamatergic synaptic network. In agreement with the previous study, we detected a developmental change in the frequency and amplitude of sEPSCs in Venus^−^ neurons in the ventromedial DR. An uncertainty that must be kept in mind is that whole-cell patch-clamp somatic recordings are only able to sample events generated at sites near the location of the recording electrode. Therefore, it is possible that the density of glutamatergic synaptic connections increases with age at distal dendritic sites, which would not be detectible due to dendritic filtering mechanisms. The fact that DR 5-HT neurons undergo significant dendritic outgrowth between P4 and P21 (Rood et al., 2014) would support the hypothesis of dendritic filtering.

In addition to the results with sEPSCs, we detected a significant increase in GABA_A_-sPSC frequency associated with a decrease in amplitude in Venus^−^ neurons at P15–P17, with respect to P5–P7. The developmental increase in the frequency of these events can be interpreted to reflect an increase in the spontaneous firing of GABAergic neurons, the probability of GABA release, and/or the number of active GABA_A_ receptor-containing synapses. However, the reductions in the decay time constants in both Venus^+^ and Venus^−^ neurons suggest changes in the subunit composition (e.g., an increase in α_1_ GABA_A_ receptor subunit expression), clustering, and/or phosphorylation state of postsynaptic receptors. Our results with DR Venus^−^ neurons are in general agreement with those of Rood et al. (2014), who reported 8-fold to 10-fold increases in IPSC frequency between P4 and P12 in the ventromedial and lateral wing DR of Pet-1-YFP mice, with a further increase in the frequency of these events at P60. Increases in sIPSC frequency during the third trimester equivalent have been detected in other neuronal populations across the brain, including cerebellar granule cells, CA3 pyramidal neurons, neurons in the CA1, and cortical pyramidal neurons (Sebe et al., 2010; Everett et al., 2012; Riebe and Hanse, 2012; Diaz et al., 2014). We refer to these currents as GABA_A_-sPSCs and not IPSCs, because, during the early stages of the third trimester equivalent developmental period, GABA_A_ receptor activation can induce membrane potential depolarization due to elevated intracellular Cl^−^ concentrations in some populations of immature neurons, including those in some brainstem nuclei (Kaila et al., 2014; Witte et al., 2014). Future experiments should determine whether GABA_A_ receptor activation exerts excitatory or inhibitory actions in DR nucleus 5-HT neurons in neonatal mice.

### Implications and future directions

This study provides additional evidence indicating that putative 5-HT DR neurons undergo significant functional maturation during the third trimester equivalent period. We further demonstrate that this is also the case for GABAergic neurons located in this brain region. Alterations in the 5-HT neurotransmitter system during development have been implicated in a variety of diseases. For instance, studies suggest that prenatal exposure to 5-HT reuptake inhibitors increases the risk of developing autism spectrum disorders (Kinast et al., 2013; Gentile, 2015). Prenatal exposure to alcohol during the rodent equivalent to the first and second trimesters of pregnancy in humans has been shown to damage developing 5-HT neurons and their axons (Eriksen and Druse, 2001; Sari and Zhou, 2004; Zhou et al., 2005). Environmental pollutants and prenatal stress have also been shown to affect immature 5-HT neurons (Cory-Slechta et al., 2008; Boix and Cauli, 2012; Glover, 2015). Therefore, future studies should investigate whether these and other insults alter the late developmental stages of this important neurotransmitter system, potentially leading to psychopathology later in life.

## Synthesis

The decision was a result of the Reviewing Editor Christophe Bernard and the peer reviewers coming together and discussing their recommendations until a consensus was reached. A fact-based synthesis statement explaining their decision and outlining what is needed to prepare a revision is listed below. The following reviewers agreed to reveal their identity: Erin Calipari, Mark Ansorge.

## References

[B1] Ali AB, Deuchars J, Pawelzik H, Thomson AM (1998) CA1 pyramidal to basket and bistratified cell EPSPs: dual intracellular recordings in rat hippocampal slices. J Physiol 507:201-217. 10.1111/j.1469-7793.1998.201bu.x 9490840 PMC2230771

[B2] Boix J, Cauli O (2012) Alteration of serotonin system by polychlorinated biphenyls exposure. Neurochem Int 60:809-816. 10.1016/j.neuint.2012.03.003 22426201

[B3] Bonnin A, Levitt P (2011) Fetal, maternal, and placental sources of serotonin and new implications for developmental programming of the brain. Neuroscience 197:1-7. 10.1016/j.neuroscience.2011.10.005 22001683 PMC3225275

[B4] Calizo LH, Akanwa A, Ma X, Pan YZ, Lemos JC, Craige C, Heemstra LA, Beck SG (2011) Raphe serotonin neurons are not homogenous: electrophysiological, morphological and neurochemical evidence. Neuropharmacology 61:524-543. 10.1016/j.neuropharm.2011.04.008 21530552 PMC3120045

[B5] Celada P, Puig MV, Casanovas JM, Guillazo G, Artigas F (2001) Control of dorsal raphe serotonergic neurons by the medial prefrontal cortex: involvement of serotonin-1A, GABA_A_, and glutamate receptors. J Neurosci 21:9917-9929. 11739599 10.1523/JNEUROSCI.21-24-09917.2001 PMC6763042

[B6] Challis C, Boulden J, Veerakumar A, Espallergues J, Vassoler FM, Pierce RC, Beck SG, Berton O (2013) Raphe GABAergic neurons mediate the acquisition of avoidance after social defeat. J Neurosci 33:13978-13988, 13988a. 10.1523/JNEUROSCI.2383-13.2013 23986235 PMC3756748

[B7] Corteen NL, Carter JA, Rudolph U, Belelli D, Lambert JJ, Swinny JD (2015) Localisation and stress-induced plasticity of GABA receptor subunits within the cellular networks of the mouse dorsal raphe nucleus. Brain Struct Funct 220:2739-2763. 24973971 10.1007/s00429-014-0824-7

[B8] Cory-Slechta DA, Virgolini MB, Rossi-George A, Thiruchelvam M, Lisek R, Weston D (2008) Lifetime consequences of combined maternal lead and stress. Basic Clin Pharmacol Toxicol 102:218-227. 10.1111/j.1742-7843.2007.00189.x 18226077

[B9] Craige CP, Lewandowski S, Kirby LG, Unterwald EM (2015) Dorsal raphe 5-HT(2C) receptor and GABA networks regulate anxiety produced by cocaine withdrawal. Neuropharmacology 93:41-51. 10.1016/j.neuropharm.2015.01.021 25656481 PMC4387096

[B10] Cummins TR, Xia Y, Haddad GG (1994) Functional properties of rat and human neocortical voltage-sensitive sodium currents. J Neurophysiol 71:1052-1064. 8201401 10.1152/jn.1994.71.3.1052

[B11] Diaz MR, Vollmer CC, Zamudio-Bulcock PA, Vollmer W, Blomquist SL, Morton RA, Everett JC, Zurek AA, Yu J, Orser BA, Valenzuela CF (2014) Repeated intermittent alcohol exposure during the third trimester-equivalent increases expression of the GABA(A) receptor δ subunit in cerebellar granule neurons and delays motor development in rats. Neuropharmacology 79:262-274. 10.1016/j.neuropharm.2013.11.020 24316160 PMC3943642

[B12] Donaldson ZR, Piel DA, Santos TL, Richardson-Jones J, Leonardo ED, Beck SG, Champagne FA, Hen R (2014) Developmental effects of serotonin 1A autoreceptors on anxiety and social behavior. Neuropsychopharmacology 39:291-302. 10.1038/npp.2013.185 23907404 PMC3870787

[B13] Eriksen JL, Druse MJ (2001) Astrocyte-mediated trophic support of developing serotonin neurons: effects of ethanol, buspirone, and S100B. Brain Res Dev Brain Res 131:9-15. 11718831 10.1016/s0165-3806(01)00240-1

[B14] Everett JC, Licón-Muñoz Y, Valenzuela CF (2012) Effects of third trimester-equivalent ethanol exposure on Cl(-) co-transporter expression, network activity, and GABAergic transmission in the CA3 hippocampal region of neonatal rats. Alcohol 46:595-601. 10.1016/j.alcohol.2012.04.003 22703993 PMC3411872

[B15] Frederick AL, Stanwood GD (2009) Drugs, biogenic amine targets and the developing brain. Dev Neurosci 31:7-22. 10.1159/000207490 19372683 PMC2786771

[B16] Gentile S (2015) Prenatal antidepressant exposure and the risk of autism spectrum disorders in children. Are we looking at the fall of Gods? J Affect Disord 182:132-137. 10.1016/j.jad.2015.04.048 25985383

[B17] Gervasoni D, Peyron C, Rampon C, Barbagli B, Chouvet G, Urbain N, Fort P, Luppi PH (2000) Role and origin of the GABAergic innervation of dorsal raphe serotonergic neurons. J Neurosci 20:4217-4225. 10818157 10.1523/JNEUROSCI.20-11-04217.2000 PMC6772634

[B18] Glover V (2015) Prenatal stress and its effects on the fetus and the child: possible underlying biological mechanisms. Adv Neurobiol 10:269-283. 10.1007/978-1-4939-1372-5_13 25287545

[B19] Gocho Y, Sakai A, Yanagawa Y, Suzuki H, Saitow F (2013) Electrophysiological and pharmacological properties of GABAergic cells in the dorsal raphe nucleus. J Physiol Sci 63:147-154. 10.1007/s12576-012-0250-7 23275149 PMC3579464

[B20] Gorter JA, Aronica E, Hack NJ, Balázs R, Wadman WJ (1995) Development of voltage-activated potassium currents in cultured cerebellar granule neurons under different growth conditions. J Neurophysiol 74:298-306. 7472332 10.1152/jn.1995.74.1.298

[B21] Gu N, Vervaeke K, Hu H, Storm JF (2005) Kv7/KCNQ/M and HCN/h, but not KCa2/SK channels, contribute to the somatic medium after-hyperpolarization and excitability control in CA1 hippocampal pyramidal cells. J Physiol 566:689-715. 10.1113/jphysiol.2005.086835 15890705 PMC1464792

[B22] Jacobs BL, Azmitia EC (1992) Structure and function of the brain serotonin system. Physiol Rev 72:165-229. 1731370 10.1152/physrev.1992.72.1.165

[B23] Kaila K, Price TJ, Payne JA, Puskarjov M, Voipio J (2014) Cation-chloride cotransporters in neuronal development, plasticity and disease. Nat Rev Neurosci 15:637-654. 10.1038/nrn3819 25234263 PMC4294553

[B24] Kapur S, Remington G (1996) Serotonin-dopamine interaction and its relevance to schizophrenia. Am J Psychiatry 153:466-476. 10.1176/ajp.153.4.466 8599393

[B25] Kinast K, Peeters D, Kolk SM, Schubert D, Homberg JR (2013) Genetic and pharmacological manipulations of the serotonergic system in early life: neurodevelopmental underpinnings of autism-related behavior. Front Cell Neurosci 7:72. 10.3389/fncel.2013.00072 23781172 PMC3679613

[B26] Kirby LG, Pernar L, Valentino RJ, Beck SG (2003) Distinguishing characteristics of serotonin and non-serotonin-containing cells in the dorsal raphe nucleus: electrophysiological and immunohistochemical studies. Neuroscience 116:669-683. 12573710 10.1016/s0306-4522(02)00584-5 PMC2832757

[B27] Kirby LG, Zeeb FD, Winstanley CA (2011) Contributions of serotonin in addiction vulnerability. Neuropharmacology 61:421-432. 10.1016/j.neuropharm.2011.03.022 21466815 PMC3110503

[B28] Kress GJ, Dowling MJ, Eisenman LN, Mennerick S (2010) Axonal sodium channel distribution shapes the depolarized action potential threshold of dentate granule neurons. Hippocampus 20:558-571. 10.1002/hipo.20667 19603521 PMC2975957

[B29] Lambe EK, Krimer LS, Goldman-Rakic PS (2000) Differential postnatal development of catecholamine and serotonin inputs to identified neurons in prefrontal cortex of rhesus monkey. J Neurosci 20:8780-8787. 11102486 10.1523/JNEUROSCI.20-23-08780.2000 PMC6773090

[B30] Lidov HG, Molliver ME (1982) An immunohistochemical study of serotonin neuron development in the rat: ascending pathways and terminal fields. Brain Res Bull 8:389-430. 6178481 10.1016/0361-9230(82)90077-6

[B31] Lin SH, Lee LT, Yang YK (2014) Serotonin and mental disorders: a concise review on molecular neuroimaging evidence. Clin Psychopharmacol Neurosci 12:196-202. 10.9758/cpn.2014.12.3.196 25598822 PMC4293164

[B60] Liu J, Morrow AL, Devaud L, Grayson DR, Lauder JM (1997) GABAA receptors mediate trophic effects of GABA on embryonic brainstem monoamine neurons in vitro. J. Neuroscience 17:2420-2428 9065503 10.1523/JNEUROSCI.17-07-02420.1997 PMC6573491

[B32] Lo Iacono L, Gross C (2008) α-Ca^2+^/calmodulin-dependent protein kinase II contributes to the developmental programming of anxiety in serotonin receptor 1A knock-out mice. J Neurosci 28:6250-6257. 10.1523/JNEUROSCI.5219-07.2008 18550767 PMC3849425

[B33] Lowery-Gionta EG, Marcinkiewcz CA, Kash TL (2015) Functional alterations in the dorsal raphe nucleus following acute and chronic ethanol exposure. Neuropsychopharmacology 40:590-600. 10.1038/npp.2014.205 25120075 PMC4289946

[B34] Madison DV, Nicoll RA (1984) Control of the repetitive discharge of rat CA 1 pyramidal neurones in vitro. J Physiol 354:319-331. 6434729 10.1113/jphysiol.1984.sp015378 PMC1193414

[B35] Migliarini S, Pacini G, Pelosi B, Lunardi G, Pasqualetti M (2013) Lack of brain serotonin affects postnatal development and serotonergic neuronal circuitry formation. Mol Psychiatry 18:1106-1118. 10.1038/mp.2012.128 23007167

[B36] Milescu LS, Yamanishi T, Ptak K, Smith JC (2010) Kinetic properties and functional dynamics of sodium channels during repetitive spiking in a slow pacemaker neuron. J Neurosci 30:12113-12127. 10.1523/JNEUROSCI.0445-10.2010 20826674 PMC2945634

[B37] Nigro MJ, Mateos-Aparicio P, Storm JF (2014) Expression and functional roles of Kv7/KCNQ/M-channels in rat medial entorhinal cortex layer II stellate cells. J Neurosci 34:6807-6812. 10.1523/JNEUROSCI.4153-13.2014 24828634 PMC6608108

[B38] Nikolaus S, Antke C, Beu M, Müller HW (2010) Cortical GABA, striatal dopamine and midbrain serotonin as the key players in compulsive and anxiety disorders–results from in vivo imaging studies. Rev Neurosci 21:119-139. 20614802 10.1515/revneuro.2010.21.2.119

[B39] Oades RD, Lasky-Su J, Christiansen H, Faraone SV, Sonuga-Barke EJ, Banaschewski T, Chen W, Anney RJ, Buitelaar JK, Ebstein RP, Franke B, Gill M, Miranda A, Roeyers H, Rothenberger A, Sergeant JA, Steinhausen HC, Taylor EA, Thompson M, Asherson P (2008) The influence of serotonin- and other genes on impulsive behavioral aggression and cognitive impulsivity in children with attention-deficit/hyperactivity disorder (ADHD): findings from a family-based association test (FBAT) analysis. Behav Brain Funct 4:48. 10.1186/1744-9081-4-48 18937842 PMC2577091

[B40] Olivier B (2015) Serotonin: a never-ending story. Eur J Pharmacol 753:2-18. 10.1016/j.ejphar.2014.10.031 25446560

[B41] Ravindran LN, Stein MB (2010) The pharmacologic treatment of anxiety disorders: a review of progress. J Clin Psychiatry 71:839-854. 10.4088/JCP.10r06218blu 20667290

[B42] Riebe I, Hanse E (2012) Development of synaptic connectivity onto interneurons in stratum radiatum in the CA1 region of the rat hippocampus. BMC Neurosci 13:14. 10.1186/1471-2202-13-14 22276909 PMC3398264

[B43] Rood BD, Calizo LH, Piel D, Spangler ZP, Campbell K, Beck SG (2014) Dorsal raphe serotonin neurons in mice: immature hyperexcitability transitions to adult state during first three postnatal weeks suggesting sensitive period for environmental perturbation. J Neurosci 34:4809-4821. 10.1523/JNEUROSCI.1498-13.2014 24695701 PMC3972713

[B44] Sari Y, Zhou FC (2004) Prenatal alcohol exposure causes long-term serotonin neuron deficit in mice. Alcohol Clin Exp Res 28:941-948. 15201637 10.1097/01.alc.0000128228.08472.39

[B45] Sebe JY, Looke-Stewart EC, Estrada RC, Baraban SC (2010) Robust tonic GABA currents can inhibit cell firing in mouse newborn neocortical pyramidal cells. Eur J Neurosci 32:1310-1318. 10.1111/j.1460-9568.2010.07373.x 20846324 PMC2956765

[B46] Stiefel KM, Englitz B, Sejnowski TJ (2013) Origin of intrinsic irregular firing in cortical interneurons. Proc Natl Acad Sci U S A 110:7886-7891. 10.1073/pnas.1305219110 23610409 PMC3651468

[B47] Storm JF (1990) Potassium currents in hippocampal pyramidal cells. Prog Brain Res 83:161-187. 2203097 10.1016/s0079-6123(08)61248-0

[B48] Suri D, Teixeira CM, Cagliostro MK, Mahadevia D, Ansorge MS (2015) Monoamine-sensitive developmental periods impacting adult emotional and cognitive behaviors. Neuropsychopharmacology 40:88-112. 10.1038/npp.2014.231 25178408 PMC4262911

[B49] Takahashi H, Nakashima S, Ohama E, Takeda S, Ikuta F (1986) Distribution of serotonin-containing cell bodies in the brainstem of the human fetus determined with immunohistochemistry using antiserotonin serum. Brain Dev 8:355-365. 3541662 10.1016/s0387-7604(86)80055-9

[B50] Vandermaelen CP, Aghajanian GK (1983) Electrophysiological and pharmacological characterization of serotonergic dorsal raphe neurons recorded extracellularly and intracellularly in rat brain slices. Brain Res 289:109-119. 6140982 10.1016/0006-8993(83)90011-2

[B51] Vitalis T, Cases O, Passemard S, Callebert J, Parnavelas JG (2007) Embryonic depletion of serotonin affects cortical development. Eur J Neurosci 26:331-344. 10.1111/j.1460-9568.2007.05661.x 17650110

[B52] Wang Y, Kakizaki T, Sakagami H, Saito K, Ebihara S, Kato M, Hirabayashi M, Saito Y, Furuya N, Yanagawa Y (2009) Fluorescent labeling of both GABAergic and glycinergic neurons in vesicular GABA transporter (VGAT)-venus transgenic mouse. Neuroscience 164:1031-1043. 10.1016/j.neuroscience.2009.09.010 19766173

[B53] Whitaker-Azmitia PM (2001) Serotonin and brain development: role in human developmental diseases. Brain Res Bull 56:479-485. 11750793 10.1016/s0361-9230(01)00615-3

[B54] Witte M, Reinert T, Dietz B, Nerlich J, Rübsamen R, Milenkovic I (2014) Depolarizing chloride gradient in developing cochlear nucleus neurons: underlying mechanism and implication for calcium signaling. Neuroscience 261:207-222. 10.1016/j.neuroscience.2013.12.050 24388924

[B55] Witteveen JS, Middelman A, van Hulten JA, Martens GJ, Homberg JR, Kolk SM (2013) Lack of serotonin reuptake during brain development alters rostral raphe-prefrontal network formation. Front Cell Neurosci 7:143. 10.3389/fncel.2013.00143 24109430 PMC3790074

[B56] Workman AD, Charvet CJ, Clancy B, Darlington RB, Finlay BL (2013) Modeling transformations of neurodevelopmental sequences across mammalian species. J Neurosci 33:7368-7383. 10.1523/JNEUROSCI.5746-12.2013 23616543 PMC3928428

[B57] Zhao S, Ting JT, Atallah HE, Qiu L, Tan J, Gloss B, Augustine GJ, Deisseroth K, Luo M, Graybiel AM, Feng G (2011) Cell type-specific channelrhodopsin-2 transgenic mice for optogenetic dissection of neural circuitry function. Nat Methods 8:745-752. 21985008 10.1038/nmeth.1668 PMC3191888

[B58] Zhou FC, Sari Y, Powrozek TA (2005) Fetal alcohol exposure reduces serotonin innervation and compromises development of the forebrain along the serotonergic pathway. Alcohol Clin Exp Res 29:141-149. 15654302 10.1097/01.alc.0000150636.19677.6f

